# Discordance between adiposity and FIB-4 risk classification in US adults

**DOI:** 10.1007/s40200-026-02007-1

**Published:** 2026-07-08

**Authors:** Luyu Xie, Diego Anazco, Azucena Herrera Chancay, Sarah E. Messiah, Jaime P. Almandoz

**Affiliations:** 1 Quantitative Biomedical Research Center, Department of Health Data Science and Biostatistics, Peter O’Donnell Jr. School of Public Health, University of Texas Southwestern Medical Center, Dallas, TX USA; 2 Department of Internal Medicine, University of Texas Southwestern Medical Center, 5323 Harry Hines Blvd, Dallas, 75390 TX USA; 3 Division of Endocrinology, Department of Internal Medicine, University of Texas Southwestern Medical Center, Dallas, TX USA; 4 Department of Epidemiology, Peter O’Donnell Jr. School of Public Health, University of Texas Southwestern Medical Center, Dallas, TX USA; 5 Department of Pediatrics, University of Texas Southwestern Medical Center, Dallas, TX USA; 6 Children’s Health System of Texas, Dallas, TX USA

**Keywords:** obesity, adiposity, fibrosis, metabolic dysfunctio-associated steatotic liver disease, body composition

## Abstract

**Purpose:**

The fibrosis-4 index (FIB-4) is a widely used noninvasive tool for fibrosis risk stratification, however, the relationship between adiposity and FIB-4-based risk classification remains unclear. We evaluated the associations between multiple measurements of adiposity and FIB-4 risk classification in a nationally representative sample of U.S. adults.

**Methods:**

We analyzed data from 10,711 adults aged 35–65 years from the 2011–2018 NHANES. Examined anthropometric measures included body mass index (BMI), total body fat percentage (TBFP), waist circumference (WC), and waist-to-hip ratio (WH). We conducted logistic regression models to evaluate the association between anthropometric measurements and high-risk FIB-4 classification and to assess whether these associations differed by age group.

**Results:**

Most participants had low-risk FIB-4 scores (72.9%), followed by intermediate-risk (25.1%) and high-risk (2%). Individuals with low FIB-4 risk classification had higher BMI, TBFP and WC (*p* < 0.01). In interaction models, the inverse associations between adiposity measures and high-risk FIB-4 classifications varied by age group. Among adults aged 51–65 years, higher BMI and TBFP were associated with lower odds of high-risk FIB-4 classification, whereas these associations were not significant for adults aged 35–50 years.

**Conclusion:**

Higher adiposity was associated with lower likelihood of high-risk FIB-4 classification. Age-stratified analyses suggested a potential discordance in the association between adiposity and FIB-4 risk classification across age groups. These findings suggest a potential discordance between adiposity-related metabolic risk and FIB-4-based risk classification, suggesting the need for age- and adiposity-informed risk assessment strategies for individuals at risk for hepatic fibrosis.

## Introduction

The prevalence of metabolic dysfunction–associated steatotic liver disease (MASLD) is increasing and projected to affect 41.4% of U.S. adults by 2050 [1]. The fibrosis-4 index (FIB-4) is a widely available, noninvasive tool recommended as the first step in guideline-based MASLD risk stratification pathways [2, 3]. Importantly, FIB-4 is a threshold-based risk stratification tool for advanced fibrosis and does not directly measure fibrosis burden.

Obesity and excess adiposity are major cardiometabolic risk factors associated with hepatic steatosis and fibrosis risk. Evidence regarding FIB-4 performance across body mass index (BMI) categories is mixed [4], with some studies suggesting reduced performance at BMI extremes, particularly in class III obesity [5, 6]. How non-BMI measures of adiposity relate to FIB-4-based risk classification remains poorly defined.

### Methods

We analyzed data from the 2011–2018 National Health and Nutrition Examination Survey (NHANES) among adults aged 35–65 years with complete laboratory data for FIB-4 calculation. This age range was selected given the reduced accuracy for the FIB-4 in individuals outside this range [7]. Standard FIB-4 cut-offs were used: <1.3 (low), 1.3–2.67 (intermediate), > 2.67 (high) [2].

Anthropometric measures included BMI, total body fat percentage (TBFP) by dual-energy X-ray absorptiometry, waist circumference (WC), and waist-to-hip ratio (WHR). TBFP was stratified by sex (≥ 25% for males, ≥ 35% for females). We used linear regression and F-tests to compare continuous adiposity measures across FIB-4 risk categories and χ² tests for categorical comparisons, with Tukey–Kramer–adjusted least-squares means for pairwise testing. To evaluate associations between adiposity measures and high-risk FIB-4 classification (FIB-4 > 2.67), we conducted separate logistic regression models for each adiposity measure incorporating age group (35–50 years vs. 51–65 years), race/ethnicity, sex, and the adiposity-by-age group interaction term. Age-group specific odds ratios were derived from the interaction models.

All analyses incorporated NHANES sampling weights and design effects and were conducted in SAS, version 9.4 (SAS Institute Inc). Two-sided *P* < 0.05 was considered statistically significant. The NHANES survey protocol was reviewed and approved by the National Center for Health Statistics Research Ethics Review Board. All participants provided written informed consent prior to participation. Because this study involved secondary analysis of publicly available, de-identified data, it was deemed exempt from review by the UTSW Medical Center IRB. This study followed the Strengthening the Reporting of Observational Studies in Epidemiology (STROBE) guidelines for observational studies.

### Results

The analysis included 10,711 adults (mean age of 49.9 years,95% CI: 49.7–50.2). The sample was evenly distributed by sex (48.5% male) and included predominantly Non-Hispanic White (65.2%) participants (Table 1). Mean FIB-4 score was 1.15 (95% CI: 1.12–1.17). Most participants (72.9%) had low FIB-4 scores (< 1.3), while 25.1% had intermediate scores (1.3–2.67) and 2.0% had high scores (> 2.67). Participants with higher FIB-4 scores were older (mean [SE] age 56.2 [0.2] -56.4 [0.5] years vs. 47.6 [0.2] years for low FIB-4, *p* < 0.001); 79.7–82.0% were aged 51–65 years compared with 36.9% in the low FIB-4 score group (*p* < 0.001). They were also more likely to be male (55.2–63.1% vs. 45.8% for low FIB-4, *p* < 0.001; Table 2).

**Table 1:** Demographics and FIB-4-based risk classifications in adults aged 35–65 years, national health and nutrition examination survey, 2011–2018.

Variable		Unweighted *N*	Mean or Weighted %		95% CI
Mean age, years		10,711	49.9		49.7 to 50.2
Sex					
Males		5,110	48.5		47.3 to 49.7
Females		5,601	51.5		50.3 to 52.7
Ethnicity and Race					
Mexican American		1,612	8.7		6.9 to 10.5
Other Hispanic		1,209	6.2		5.0 to 7.5
Non-Hispanic White		3,557	65.2		61.6 to 68.9
Non-Hispanic Black		2,486	10.9		9.1 to 12.8
Non-Hispanic Asian		1,476	5.4		4.4 to 6.5
Other/multi-race		371	3.4		2.8 to 4.1
FIB-4^a^					
Mean		10,711	1.15		1.12 to 1.17
< 1.3		7,782	72.9		71.4 to 74.5
1.3–2.67		2,699	25.1		23.6 to 26.6
> 2.67		230	2.0		1.6 to 2.3

a FIB-4 is measured by (Age [years] x AST [U/L]) / (Platelet count [109/L] x √ALT [U/L])

Paradoxically, participants with low FIB-4 had greater adiposity, including higher mean BMI (30.3 [0.1] vs. 28.7 [0.2] and 28.4 [0.6] kg/m²; *p* < 0.001), TBFP (34.4 [0.2] % vs. 32.2 [0.3] % and 30.4 [1.6] %; *p* < 0.001), and WC (102.1 [0.3] cm vs. 100.3 [0.5] cm; *p* = 0.006) compared with intermediate and high FIB-4 groups (Table 2). Together, these findings suggest that obesity and central adiposity may be associated with lower FIB-4-based risk classification, highlighting potential classification differences in individuals with high-adiposity.

**Table 2:** Demographics and anthropometric Measures by FIB-4- based risk classification, national health and nutrition examination survey, 2011–2018

FIB-4	< 1.3	1.3–2.67	> 2.67	*P*-value^a^
N (weighted %)	7,782 (72.9)	2,699 (25.1)	230 (2.0)	
Age, mean (SE), y	47.6 (0.2)	56.2 (0.2)	56.4 (0.5)	< 0.001
35–40	1,909 (24.8)	76 (2.2)	12 (4.1)	< 0.001
41–45	1,531 (19.9)	158 (5.8)	11 (4.4)	
46–50	1,344 (18.4)	286 (12.3)	14 (9.5)	
51–55	1,223 (16.1)	493 (19.4)	38 (17.5)	
56–60	950 (12.7)	681 (27.9)	70 (34.4)	
60–65	825 (8.1)	1,005 (32.4)	85 (30.1)	
Sex, n (%)				< 0.001
Male	3,436 (45.8)	1,529 (55.2)	145 (63.1)	
Female	4,346 (54.2)	1,170 (44.8)	85 (36.9)	
Ethnicity and Race, n (%)				< 0.001
Mexican American	1,236 (9.8)	343 (5.5)	33 (8.2)	
Other Hispanic	894 (6.9)	288 (4.4)	27 (4.6)	
Non-Hispanic White	2,591 (63.0)	892 (71.3)	74 (68.5)	
Non-Hispanic Black	1,703 (11.0)	712 (10.7)	71 (12.8)	
Non-Hispanic Asian	1,072 (5.6)	387 (5.2)	17 (3.1)	
Other/multi-race	286 (3.7)	77 (2.9)	8 (2.6)	
Anthropometric measures				
BMI, mean (SE), kg/m^2^	30.3 (0.1)* ^†^	28.7 (0.2)*	28.4 (0.6) ^†^	< 0.001
TBFP, mean (SE)	34.4 (0.2)* ^†^	32.2 (0.3)*	30.4 (1.6) ^†^	< 0.001
WC, mean (SE), cm	102.1 (0.3)*	100.3 (0.5)*	100.7 (1.8)	0.006
WHR, mean (SE)	0.94 (0.00)	0.94 (0.01)	0.95 (0.02)	0.967

^a^ Survey-weighted linear regression and F-test was used for continuous variables, and survey-weighted chi-square analysis was used for categorical variables

*^†^ *P* < 0.05, Pairwise comparisons were conducted using least squares means with Tukey-Kramer adjustment for multiple comparisons

BMI (body mass index), *TBFP* (total body fat percentage) *WC* (waist circumference), *WHR* (waist to hip ratio)

Additional age-stratified analyses indicated that the inverse associations between adiposity measures and high FIB-4 risk classification were more pronounced among adults aged 51–65 years than among those aged 35–50 years (Table 3). Specifically, among participants aged 51–65 years, higher BMI (adjusted OR 0.82, 95% CI 0.68–0.99) and total body fat percentage (TBFP) (adjusted OR 0.62, 95% CI 0.42–0.92) were associated with lower odds of high-risk FIB-4 classification. In contrast, these associations were not statistically significant among adults aged 35–50 years. Interaction testing revealed a borderline significant interaction between age and TBFP (*p* = 0.056). Together, these findings suggest a possible age-related pattern in the association between adiposity and FIB-4–based risk classification.

**Table 3:** Age-stratified associations between adiposity measures and high-risk FIB-4 classification

Measure	Age group	Adjusted OR (95% CI)^a^	*P* value^a^	*P* for interaction^a^
BMI (per 5 kg/m^2^)	35–50	0.85 (0.55–1.30)	0.431	0.884
	51–65	0.82 (0.68–0.99)	0.039	
TBFP (per 5%)	35–50	0.96 (0.68–1.35)	0.792	0.056
	51–65	0.62 (0.42–0.92)	0.018	
WC (per 10 cm)	35–50	0.81 (0.63–1.03)	0.089	0.428
	51–65	0.90 (0.76–1.07)	0.244	
WHR (per 0.1)	35–50	0.49 (0.08–2.82)	0.397	0.771
	51–65	0.62 (0.18–2.06)	0.407	

^**a**^ Each adiposity measure was evaluated in a separate logistic regression model including the adiposity measure, age group (35–50 vs. 51–65 years), adiposity measure × age group interaction, sex, and race/ethnicity. Age-group-specific ORs were derived from the interaction model. The P value for interaction tests whether the adiposity–high-risk FIB-4 association differed by age group. BMI (body mass index), *TBFP* (total body fat percentage), *WC* (waist circumference), *WHR* (waist to hip ratio)

## Discussion

In this nationally representative sample, higher adiposity was associated with lower likelihood of high-risk FIB-4 classification, in contrasts with the established relationship between excess adiposity and risk of hepatic steatosis and fibrosis [8]. However, it is important to highlight that our findings relate specifically to FIB-4 based risk classification rather than confirmed hepatic fibrosis. Interaction analyses suggested potential age-related differences in the association between adiposity and high-risk FIB-4 classification. As a result, younger adults may be less likely to meet the threshold for a high-risk classification, potentially limiting the extent to which adiposity-related risk is captured by FIB-4 alone. However, since age is directly incorporated into the FIB-4 equation, a part of the observed association might be a result of the properties of the score itself rather than an underlying biological relationship.

These findings suggest that low FIB-4 classifications may not fully capture adiposity-related metabolic risk in some individuals. Incorporating adiposity-related measures into risk assessment strategies may improve the accuracy of noninvasive fibrosis risk stratification [6, 9, 10]. Prior work has demonstrated that incorporating WC improves FIB-4 performance for detecting advanced fibrosis by VCTE [11]. Moreover, anthropometric measures such as WC have outperformed BMI and other indices in predicting steatosis and increased liver stiffness in population-based analyses. Taken together, these data suggest that adiposity measures may complement existing fibrosis risk stratification pathways [12].

This analysis was conducted in a general population sample and was not restricted to individuals meeting formal MASLD criteria, as a result, these findings should not be directly interpreted as reflecting the performance among patients with confirmed MASLD. Accordingly, adiposity measures were examined as correlates of metabolic risk rather than diagnostic surrogates of hepatic steatosis or fibrosis. FIB-4 is a risk stratification tool for advanced fibrosis and does not quantify fibrosis burden. Therefore, these findings reflect patterns of risk classification rather than histologic or elastography-confirmed fibrosis. In addition, other factors such as diabetes and alcohol consumption, which are independently associated with hepatic steatosis and fibrosis were not included in our regression analyses since the objective was to assess the overall associations between adiposity measures and FIB-4 classification in a general population rather than estimate for independent causal effects. However, given the central role of FIB-4 in contemporary MASLD care pathways, these results provide important insight into real-world risk stratification [3]. In addition, only a small proportion of participants were in the high-risk FIB-4 classification group (> 2.67), which might limit the precision of interaction estimates. Longitudinal studies incorporating advanced noninvasive testing or histology-based assessments will be essential to clarify the clinical consequences of this observed discordance.

In conclusion, our findings showed that greater adiposity was associated with lower FIB-4-based risk classification. These findings suggest a potential age-related discordance between adiposity and FIB-4-based risk classification and suggest that reliance on FIB-4 alone may result in lower fibrosis risk stratification among individuals with high adiposity despite substantial metabolic risk. As risk stratification pathways increasingly guide referral decisions and resource allocation, understanding age- and adiposity-related classification dynamics is clinically important. Given the high and rising prevalence of MASLD and obesity, improved risk stratification approaches that are simple, reproducible, and equitable are urgently needed. Future research should evaluate age-aware and adiposity-informed strategies across diverse populations and care settings.

## Data Availability

The data analyzed in this study are publicly available through the National Health and Nutrition Examination Survey (NHANES) repository.

## Abbreviations

MASLD: metabolic dysfunction–associated steatotic liver disease
FIB-4: fibrosis-4 index
BMI: body mass index
TBFP: total body fat percentage
WC: waist circumference
WHR: waist-to-hip ratio
NHANES: National Health and Nutrition Examination Survey
VCTE: vibration-controlled transient elastography
